# Health systems as venture capital investors in digital health: 2011–2019

**DOI:** 10.1038/s41746-020-00311-5

**Published:** 2020-08-04

**Authors:** Kyan C. Safavi, Adam B. Cohen, David Y. Ting, Sreekanth Chaguturu, Jack S. Rowe

**Affiliations:** 1 Massachusetts General Physicians Organization, Boston, MA USA; 2 Harvard Medical School, Boston, MA USA; 3 The Johns Hopkins University Applied Physics Lab, Laurel, MD USA; 4 The Johns Hopkins Hospital, Baltimore, MD USA; 5 CVS Health, Woonsocket, RI USA

**Keywords:** Technology, Health care economics

## Abstract

Provider health systems as venture capital investors in digital health are uniquely positioned in the industry. Little is known about the volume or characteristics of their investments and how these compare to other investors. From 2011 to 2019, we found that health systems made 184 investments in 105 companies. Compared with other investors, they were more likely to invest in companies focused on workflow, on-demand health services, and data infrastructure/interoperability.

## Introduction

Advancements in digital health promise to disrupt healthcare by enabling greater connectivity between patients and providers, expanding the scope and utility of health data, improving the patient experience, and reducing waste^1,2^. Growth in digital health has been bolstered by investment capital firms, which have provided much of the financing for new companies. Over the past decade, billions of dollars have been invested into the sector with a 400% increase in investment deals during this period^3^. Through their investment choices, investment capital firms have attempted to shape the future of digital healthcare innovation. Recently, a growing body of literature has sought to evaluate how private sector investments are shaping our broader healthcare landscape^4–7^.

Health systems have recently joined the massive digital health investing trend. Venture capital funds managed by health systems are in a unique position. For their institutions, they offer the promise of both financial returns and strategic positioning by supporting companies that build solutions to address clinical and operational challenges. Compared with other investors, health systems can provide unique value to companies as they hold domain expertise and direct access to both providers and patients^8,9^. Their live clinical environments can be used for product testing and development. Thus health systems can impact the companies that they invest in as simultaneous financers, co-developers, and future customers.

Unique conflicts of interest may also exist. Health systems must be wary of giving companies in which they have a financial interest access to patients and their data without appropriate regard to data privacy, ownership, governance, and security. They must be cautious of encouraging patients to use digital services in which they have a vested financial interest.

Despite their unique position, little is known about health system venture investment into digital health companies and how their activity compares with other digital health investors. We describe patterns among health system investors, including the volume of investments, the types of products and services that they invested in, and how their investments compared to other investors in digital health.

## Results

### Investment volume and characteristics

During the analysis period, there were 1629 investors with 4533 investments across 1215 companies, with an aggregate investment of $33.0 billion. Among investors, 55 (3.4%) were health systems. They accounted for 184 (4.1%) investments and invested in 105 (8.6%) of companies. Health systems participated in investment deals that totaled $2.1 billion. The number of investments made by other investor types included: traditional venture capital 2855 (65.7%), corporate venture capital 590 (13.6%), other 391 (9.0%), private equity 215 (4.9%), angel groups 95 (2.2%), accelerators and incubators 93 (2.1%), hedge funds or asset managers 76 (1.7%), and foundations or impact investors 31 (0.7%).

**Fig. 1:**
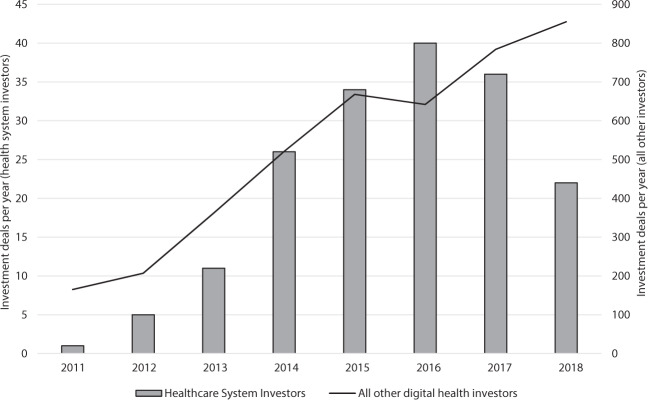
Yearly volume of digital health investment deals by health systems. Number of digital health investments per year by health system investors and all other investors.

**Fig. 2:**
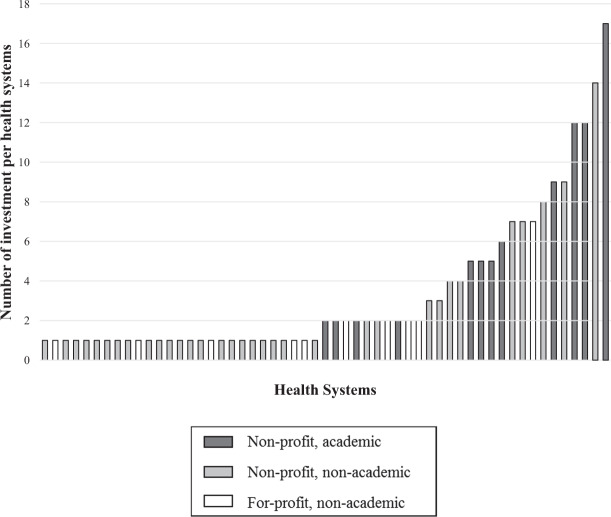
Volume of digital health investment deals by individual health systems. Total volume of investments in digital health companies by individual health systems.

Figure 1 demonstrates the number of investments by health systems and all other digital health investors from 2011 to 2018, with 2019 omitted as it is a partial year of data. Among all health system investors, 20 (36.4%) were associated with academic health systems. Among all health systems investors, 45 (81.8%) were associated with not-for-profit systems. The top 10 health systems with the most investments during the study period accounted for 102 (55.4%) of all investments made by health systems (Fig. 2).

Characteristics of digital health products and services investments by health systems are reported in Table 1 and compared to investments made by all other digital health investors. Most investments made by health systems were in companies that remained active as of March 31, 2019 (86.4%), were acquired by larger companies (10.9%), or had undergone an initial public offering (0.5%). Only 2.2% of companies were defunct. These outcomes were similar to other investors (*p* > 0.05 in all comparisons).

**Table 1:** Characteristics of digital health products and services invested in by health systems versus all other digital health investors.

	Investments by healthcare systems (*n* = 184)	Investments by all other digital health investors (*n* = 4349)	Difference (%)	*p* Value
Technology focus				
Non-clinical workflow	16.3%	8.4%	7.9%	0.0007*
Clinical workflow	12.0%	5.5%	6.4%	0.001*
On-demand healthcare services	12.0%	7.4%	4.6%	0.0316*
Data infrastructure and interoperability	10.3%	4.5%	5.8%	0.0011*
Consumer health information	7.6%	9.4%	−1.8%	0.5168
Monitoring of disease	7.6%	6.4%	1.2%	0.5392
Clinical decision support and precision medicine	6.0%	3.3%	2.7%	0.0575
Customer acquisition and relationship management	5.4%	3.9%	1.5%	0.3302
Care coordination	4.3%	4.5%	−0.2%	1
Research and development catalyst	3.8%	7.7%	−3.9%	0.0613
Patient adherence	3.3%	2.3%	0.9%	0.4516
Treatment of disease	2.7%	5.1%	−2.3%	0.2211
Diagnosis of disease	2.2%	3.7%	−1.5%	0.4165
Fitness and wellness	2.2%	12.3%	−10.1%	0*
Population health management	2.2%	4.2%	−2.1%	0.2528
Medical reference / education	1.1%	0.6%	0.4%	0.3456
Marketplace	0.5%	3.8%	−3.3%	0.0145*
Prevention of disease	0.5%	0.8%	−0.3%	1
Health benefits administration	0.0%	4.5%	−4.5%	0.0006*
Other	0.0%	1.6%	−1.6%	0.1158
Technology type				
No specific technology type	42.9%	45.3%	−2.3%	0.5462
Artificial intelligence	13.0%	13.5%	−0.5%	1
Telemedicine	12.0%	10.9%	1.1%	0.6293
Genomics and sequencing	8.7%	4.7%	4.0%	0.0206*
Digital medical device	7.1%	5.1%	2.0%	0.2335
Wearables and biosensors	7.1%	10.0%	−2.9%	0.2539
Remote monitoring	5.4%	2.7%	2.7%	0.0401*
Non−medical device hardware	2.7%	1.6%	1.1%	0.2258
Augmented and virtual reality	0.5%	1.4%	−0.9%	0.5184
Other	0.5%	2.1%	−1.6%	0.1844
Blockchain	0.0%	1.1%	−1.1%	0.262
Internet of things	0.0%	0.8%	−0.8%	0.4011
Robotics	0.0%	1.0%	−1.0%	0.416
End user				
Providers	70.1%	36.7%	33.4%	0*
Individual consumers	20.7%	34.0%	−13.3%	0.0001*
Employers and employees	4.9%	12.5%	−7.6%	0.0011*
Biopharma companies	2.2%	7.1%	−4.9%	0.0067*
Other	1.1%	4.7%	−3.6%	0.017*
Payers	1.1%	4.1%	−3.0%	0.0493*
Medical device companies	0.0%	0.4%	−0.4%	1
Pharmacies	0.0%	0.7%	−0.7%	0.6329
Customer				
Providers	79.9%	42.5%	37.4%	0*
Individual consumers	7.6%	21.7%	−14.1%	0*
Employers	5.4%	12.6%	−7.1%	0.0026*
Payers	3.3%	9.0%	−5.8%	0.0048*
Biopharma companies	2.2%	7.7%	−5.5%	0.0024*
Other	1.6%	5.6%	−4.0%	0.0183*
Medical device companies	0.0%	0.4%	−0.4%	1
Pharmacies	0.0%	0.6%	−0.6%	0.6234

* indicates a statistically significant difference.

## Discussion

The financing of digital health companies represents a new effort in which health systems are seeking to shape the future of healthcare—one with complex incentives and boundaries. We describe key ways in which health systems are acting as a small but important group of investors in digital health companies. Over the past 8 years, health systems made 184 investments in 105 early stage digital health companies. Over one-third of health systems making these investments were academic and the majority were non-profit organizations.

Health system investors have multiple motivations that could influence their investment decisions, including funding solutions that align with institutional priorities, generating non-clinical revenue, commercializing internally developed intellectual property, and gaining insight into upcoming and potentially disruptive innovations to maintain organizational competitiveness^8,11^. Compared to other digital health investors, we found that health systems are making unique investment choices. Health systems were more likely to invest in companies with healthcare providers as the end user and/or customer and in companies that focused on clinical and non-clinical workflow solutions, on-demand healthcare services, and data infrastructure/interoperability. In doing so, health systems appear to be focusing their investments on what they know—the provision of healthcare services^8,12^. The tendency to invest in these areas may also reflect that health systems perceive them as particularly promising, sustainable, and scalable. Conversely, health systems were less likely to invest in fitness and wellness products, which was the top investment area for other investors. Health systems may not see these products as impactful, profitable, or aligned with their mission.

In contrast to the broader digital health investor landscape, we observed a decline in the total volume of digital health investments by health system investors in 2017 and 2018^3,7^. The reasons for this are unclear and warrant further investigation. Further, the magnitude and types of future investments in digital health by all investor types may be dramatically impacted by the coronavirus disease-2019 pandemic.

There is value in further monitoring and evaluation of investments made by health systems, as these choices have implications for patient, provider, industry, and health policy stakeholders. First, the products and services in which health systems invest may indicate their prediction of which will generate value in healthcare and meaningfully impact patients and/or providers. Second, an improved understanding of best practices and the strategic and financial returns on investment achieved by health systems would better inform how health systems may use these activities to support their core mission. Third, health system investments should be monitored due to the potential conflicts of interests that they represent for the institution. Public reporting standards and disclosure requirements would help ensure that these potential conflicts are appropriately acknowledged and managed. It is uncertain the degree to which such policies have been established within health systems amidst the recent flood of digital investments.

This study has several limitations. The database captures publicly disclosed investment deals >$2 million as deals of lesser value are not consistently filed with the U.S. Securities and Exchange Commission (SEC) or announced publicly by the company or the investor. Less than 7% of digital health deals are estimated to be <$2 million, but these deals are not included in our study^10^. This represents a potential source of bias. Also, the database includes companies that are venture-backed such that large, well-established companies with a portion of their work in digital health are not captured. Further, while the database used in this study is thought to be comprehensive, it is difficult to independently verify whether every investment deal >$2 million is captured; though, we are not aware of a more comprehensive database of investments in digital health companies.

The emerging trend among health systems to invest in venture-backed digital health companies represents a new frontier in their ability to influence the future of healthcare. This activity warrants continued monitoring and further exploration of its motivations and outcomes.

## Methods

### Study cohort and database

We performed a cross-sectional analysis from January 1, 2011 through March 31, 2019 using the Digital Health Funding Database maintained by Rock Health^10^. The database contains a repository of U.S.-based venture-backed digital health (regardless of size) with a disclosed investment deal of at least $2 million U.S. dollars (USD) and collects investment information through quarterly reviews of filings to the SEC and public announcements by companies and their investors. Funding transactions <$2 million USD are not consistently filed in the public domain and were excluded. Additional data on companies included the defining technology type(s) of services produced, technology focus, end user type, customer type, and company status. Further details on the methodology of identifying and categorizing deals in this database are publicly available^10^.

Investors were subcategorized as either health systems or all other digital health investors, which included traditional venture capital, corporate venture capital (excluding health systems), private equity, angel groups, accelerators and incubators, hedge funds or asset managers, foundations or impact investors, and other.

### Statistical analysis

We calculated the number of investment deals and the total amount of USD invested by health systems and all other categories of investors during the study period. Further, we calculated the number of deals involving health systems by company status. In addition, we compared the proportion of investments made by health systems to all other digital health investors in terms of technology focus, technology type, end user type, customer type, and company status. We used Fischer Exact tests to assess statistical differences. A *p* value of < 0.05 was considered statistically significant in all cases.

### Reporting summary

Further information on research design is available in the Nature Research Reporting Summary linked to this article.

## Supplementary information

Reporting Summary

## Data Availability

The datasets generated during and/or analyzed during the current study may be requested from the corresponding author.
