# Supplementary material for: Health systems as venture capital investors in digital health: 2011–2019
Source: NPJ Digit Med. 2020 Aug 4;3:103. doi: 10.1038/s41746-020-00311-5 (PMC7403411; doi:10.1038/s41746-020-00311-5)
Supplement: Supplementary file 1 — Reporting Summary [file 41746_2020_311_MOESM1_ESM.pdf]

nature research

Corresponding author(s): Kyan Cyrus Safavi

Last updated by author(s): Jun 10, 2020

Reporting Summary

Nature Research wishes to improve the reproducibility of the work that we publish. This form provides structure for consistency and transparency in reporting. For further information on Nature Research policies, see our Editorial Policies and the Editorial Policy Checklist.

Statistics

For all statistical analyses, confirm that the following items are present in the figure legend, table legend, main text, or Methods section.

n/a	Confirmed	
☐	☒	The exact sample size (n) for each experimental group/condition, given as a discrete number and unit of measurement
☐	☒	A statement on whether measurements were taken from distinct samples or whether the same sample was measured repeatedly
☐	☒	The statistical test(s) used AND whether they are one- or two-sided<br>*Only common tests should be described solely by name; describe more complex techniques in the Methods section.*
☐	☒	A description of all covariates tested
☐	☒	A description of any assumptions or corrections, such as tests of normality and adjustment for multiple comparisons
☐	☒	A full description of the statistical parameters including central tendency (e.g. means) or other basic estimates (e.g. regression coefficient) AND variation (e.g. standard deviation) or associated estimates of uncertainty (e.g. confidence intervals)
☐	☒	For null hypothesis testing, the test statistic (e.g. F, t, r) with confidence intervals, effect sizes, degrees of freedom and P value noted<br>*Give P values as exact values whenever suitable.*
☐	☒	For Bayesian analysis, information on the choice of priors and Markov chain Monte Carlo settings
☐	☒	For hierarchical and complex designs, identification of the appropriate level for tests and full reporting of outcomes
☐	☒	Estimates of effect sizes (e.g. Cohen's d, Pearson's r), indicating how they were calculated

Our web collection on statistics for biologists contains articles on many of the points above.

Software and code

Policy information about availability of computer code

Data collection	No software was used
Data analysis	No software was used

For manuscripts utilizing custom algorithms or software that are central to the research but not yet described in published literature, software must be made available to editors and reviewers. We strongly encourage code deposition in a community repository (e.g. GitHub). See the Nature Research guidelines for submitting code & software for further information.

Data

Policy information about availability of data

All manuscripts must include a data availability statement. This statement should provide the following information, where applicable:
- Accession codes, unique identifiers, or web links for publicly available datasets
- A list of figures that have associated raw data
- A description of any restrictions on data availability

The datasets generated during and/or analyzed during the current study may be requested from the corresponding author. The data used in this study is not publicly available. Figure 1, Figure 2, and Table 1 are derived based upon data from the corresponding author's dataset.

Field-specific reporting

Behavioural & social sciences study design

All studies must disclose on these points even when the disclosure is negative.

Study description	We performed a cross-sectional analysis from January 1, 2011 through March 31, 2019 of the digital health industry. We used quantitative data.
Research sample	The research sample is collected and maintained by Rock Health in the Digital Health Funding Database. It includes U.S.-based venture-backed digital health (regardless of size) with a disclosed an investment deal of at least $2 million U.S. dollars (USD). The sample is derived based upon investment information data collected through quarterly reviews of filings to the U.S. Securities and Exchange Commission (SEC) and public announcements by companies and their investors. We believe that the sample is respresentative of US-based venture-backed digital health companies. Companies with smaller funding amounts are not within the sample as their financial filings are not consistently disclosed. This is a potential source of bias that is noted in the discussion section.
Sampling strategy	This was a cross-sectional descriptive study based upon the research sample above.
Data collection	Data collection was performed by Rock Health. Further details on the methodology of identifying and categorizing deals in this database are publicly available. https://rockhealth.com/data/funding-raw-data
Timing	January 1, 2011 through March 31, 2019
Data exclusions	No data were excluded from the analysis
Non-participation	Not applicable to our research study
Randomization	Not applicable to our research study

Reporting for specific materials, systems and methods

We require information from authors about some types of materials, experimental systems and methods used in many studies. Here, indicate whether each material, system or method listed is relevant to your study. If you are not sure if a list item applies to your research, read the appropriate section before selecting a response.

Materials & experimental systems

n/a	Involved in the study	
☒	☐	Antibodies
☒	☐	Eukaryotic cell lines
☒	☐	Palaeontology and archaeology
☒	☐	Animals and other organisms
☒	☐	Human research participants
☒	☐	Clinical data
☒	☐	Dual use research of concern

Methods

n/a	Involved in the study	
☒	☐	ChIP-seq
☒	☐	Flow cytometry
☒	☐	MRI-based neuroimaging
